# Supplementary material for: Using spatio-temporal graph neural networks to estimate fleet-wide photovoltaic performance degradation patterns
Source: PLoS One. 2024 Feb 14;19(2):e0297445. doi: 10.1371/journal.pone.0297445 (PMC10866472; doi:10.1371/journal.pone.0297445)
Supplement: Attachment — Submitted filename: 2310_decomposition_reviewer_response.pdf [file pone.0297445.s001.pdf]

Revisions to Manuscript

Oct 17, 2023

Dear Reviewers,

Thank you for the careful reviews of our manuscript. We appreciate the responses and have addressed the comments below from each of the reviewers. The reviewer's comments are in italics and our responses are below indented below in normal text. We have provided an annotated copy of the manuscript with the resulting changes to the text. The second round of revisions are highlighted in red text in the annotated manuscript.

Reviewer 1:

The authors have mentioned PLR in the 'Introduction' section of the manuscript which is supported by reference [5] in the manuscript. The author must express the formula for PLR in the present case as expressed by Deceglie et al.

> The authors have reviewed the manuscript suggested by Reviewer #1 and feel like the equation provided in this manuscript (Eq 7) is equivalent to the one expressed by Deceglie et al.

What is POA in 6K model?

> We have updated our manuscript to define the Plane of Array (POA) Irradiance in the body of the text.

The authors have used several acronyms in the manuscript which need to be defined in the manuscript.

> The authors have read through the manuscript again and added any undefined acronyms

MAPE(%) is found least for PV-stGNN-PLR in all the three discussed cases in the manuscript. However, this loss function of PV-stGNN-PLR consists of a global reconstruction error with additional constraints applied individually to the fluctuation and aging terms. Is this is the reason for achieving low MAPE(%). Please justify!

Yes, our carefully designed loss function ensures that PV-stGNN-PLR can accurately extract the complex (non-linear and/or non-monotonic) performance loss patterns of PV systems from aging terms. The fluctuation terms extract multi-level seasonal patterns for each PV system constrained by the topological information captured by the spatiotemporal graph and global reconstruction error guarantees that the extracted aging terms match with corresponding PV systems.

Since your data contains seasonality, did you try Holt-winters exponential smoothing? It will be great to handle the time series data containing a seasonality.

No, holt-winters exponential smoothing can only be used to extract linear, linear with damping, and exponential trend [1]. Holt-Winters is another commonly applied model for PLR estimation, but was not implemented in the scope of this study. It fails to capture the more complex non-linear and/or non-monotonic performance loss patterns in PV systems.However, our design PV-stGNN-PLR, an unsupervised machine learning method, can learn the complex non-linear PLR of PV systems.

[1]: Kalekar, Prajakta S. "Time series forecasting using holt-winters exponential smoothing." Kanwal Rekhi school of information Technology 4329008.13 (2004): 1-13.

Can you please explain why did you choose Euclidean distance and MAPE to evaluate PLR estimation errors? Did you consider any other Time Series Error metrics?

Yes, other metrics MAE, MSE, and RMSE are also found in literature to report PLR estimation errors. However, these values are in same units as the original data, which makes them harder to compare the accuracy across data with different scales. MAPE, on the other hand, express the error as a percentage of actual values making it easier to compare prediction errors across PV systems. Hence, if MAPE is 1.5%, this means the average prediction error is 1.5% of actual values. Euclidean distance measures spatial separation between RDP (real degradation patterns) and EDP (estimated degradation patterns) which are both sequences and can be naturally considered as two data points in a high-dimension space. We have updated the methodology with some clarifications of Euclidean Distance and its importance in our model.

The alignment of the text in the manuscript is not justified.

The authors have used the supplied manuscript template provided by PLOSone. We have not edited the document setup parameters.

Define ‘mqlj’ in the equation (3).

The authors have clarified the element M_{qlj} in the body of the manuscript.

There are several grammatical errors in the manuscript.

The authors have read through the manuscript and checked for grammatical errors, correcting them as fit.

References are randomly collected without any standard format style such as APA, Chicago. MLA, etc.

The authors have followed the instructions on PLOSone guidelines on the formatting of the references. They are formatted correctly as per the instructions on PLOSone.

Reviewer #2

I really appreciate the research carried out in the proposed article and I wish to see it published. The paper is very well written and presented. I would be glad to see your future publications in this field.

The authors appreciate the work reviewers due to ensure a quality submission.
